# Sexual maturation and smoltification in domesticated Atlantic salmon (*Salmo salar* L.) – is there a developmental conflict?

**DOI:** 10.14814/phy2.13809

**Published:** 2018-09-02

**Authors:** Per Gunnar Fjelldal, Rüdiger Schulz, Tom O. Nilsen, Eva Andersson, Birgitta Norberg, Tom Johnny Hansen

**Affiliations:** ^1^ Institute of Marine research (IMR) Matre Aquaculture Research Station Matredal Norway; ^2^ Institute of Marine research (IMR) Nordnes Bergen Norway; ^3^ Reproductive Biology Group Division Developmental Biology Department of Biology Faculty of Sciences Utrecht University Utrecht The Netherlands; ^4^ Uni Research Environment Bergen Norway; ^5^ Institute of Marine research (IMR) Austevoll Research Station Storebø Norway

**Keywords:** Phenotype, physiology, sexual maturation, smoltification

## Abstract

We present data from two experiments that examined how the developmental processes of smoltification and sexual maturation proceed in parallel in domesticated Atlantic salmon. Onset of maturation and smoltification was stimulated using temperature and photoperiod. Our observations on gonadosomatic index (GSI), spermatogenic activity, gill Na+, K+‐ATPase enzyme (NKA) activity, and plasma 11‐ketotestosterone (11‐KT), Na, Cl, and Ca show that smoltification and maturation were both triggered and developed in parallel in male Atlantic salmon, but that the progressing maturation impaired hypoosmoregulation. Female maturation started after completion of smoltification. Furthermore, we present data showing that domesticated salmon can physiologically smoltify–desmoltify–resmoltify within a short period of time, and that development of a secondary sexual characteristic, such as a kype, depends on size in male postsmolts.

## Introduction

In its habitats in the north Atlantic region, wild Atlantic salmon (*Salmo salar* L.) is exposed to marked seasonal changes in both photoperiod and temperature. The anadromous salmon hatches and spends 1–6 years in freshwater before migrating to the sea during the spring, where most of the somatic growth takes place. The physiological adaptions allowing the salmon to survive and grow in seawater are called smoltification or parr‐smolt transformation (Hoar 1988). After a somatic growth period of 1–3 years, salmon migrate back to their native river for sexual maturation and spawning (Metcalfe and Thorpe 1990; Klemetsen et al. 2003). In addition to this mode of maturation following at least 1 year in the sea, males can mature as parr (Myers et al. 1986; Berrill et al. 2003; Skilbrei and Heino 2011), or in some cases first as parr and then as adults (Mitans 1973; Thorpe and Morgan 1980). Both smoltification and sexual maturation involve dramatic changes in physiology, behavior, and morphology. One important change associated with smoltification is to develop the hypo‐osmoregulatory ability as preparation for a marine life (Hoar 1988). The gill is an important organ in this regard, and gill Na^+^, K^+^‐ATPase enzyme (NKA) activity increases during smoltification and is often further elevated after seawater entry. Elevated gill NKA activity is associated with increased expression of SW‐type (*α*1b) and decrease in FW‐type (*α*1a) catalytic *nka* subunits (Nilsen et al. 2003, 2007). High NKA activity correlates well with hypo‐osmoregulatory ability, and allows smolts to enter seawater with minimum osmotic disturbance (Hoar 1988). An important change associated with sexual maturation in salmon is the activation of the brain–pituitary system. The elevated production and release of follicle stimulating hormone (Fsh) from the pituitary then stimulates gonadal steroid hormone production, and downstream effects of Fsh and sex steroids promote germ cell development (Taranger et al. 2010). In males, 11‐ketotestosterone plasma concentrations are a good indicator of the maturational status in several salmonid species, including Atlantic salmon. In females, hepatic synthesis of the yolk protein precursor vitellogenin is induced by estrogens and is an indicator of activation of the reproductive system in females (Tyler et al. 1990). There is a close correlation between total plasma calcium and plasma vitellogenin levels (Elliot et al. 1979; Norberg et al. 1989).

The debate regarding the question if smoltification and maturation are mutually exclusive developmental processes has a history of decades (Evropeizeva 1960; Thorpe 1986, 1994; Thorpe and Metcalfe 1998). Thorpe (1986) concluded that while maturation and smolting are not mutually exclusive, they are in developmental conflict. Indeed, there is no report documenting that the two developmental processes co‐occur in wild Atlantic salmon, in contrast to wild Chinook salmon (*Oncorhynchus tshawytscha*) where smoltification and downstream migration were reported in males (minijacks) that had already started pubertal development (Larsen et al. 2010). In farmed Atlantic salmon, however, breeding (Gjedrem 2000), optimization of dietary composition and energy level (Hillestad and Johnsen 1994), use of artificial photoperiods (Hansen et al. 1992), and temperature manipulation (Siemien and Carline 1991) have increased somatic growth tremendously, which may contribute to smoltification and maturation occurring at the same time in males (Fjelldal et al. 2011). There is, however, no information on how maturation and smoltification codevelop in salmon males, nor when and triggered by what signals maturation starts during smolt development. With the aquaculture industry's present activities to produce large smolts or postsmolts in closed systems, such knowledge becomes highly relevant. This change in production practice aims at avoiding sea lice (*Lepeophtheirus salmonis*) infestation (Taranger et al. 2015), and involves the use of different flow‐through or recirculating systems. Common to all strategies is the elevation of water temperature compared to traditional farming. However, both temperature (Fjelldal et al. 2011; Imsland et al. 2014), and size (Fjelldal et al. 2007) are important factors contributing to the risk of postsmolt maturation in male Atlantic salmon. Early, unwanted sexual maturation in seawater is a serious animal welfare issue due to compromised health, problems with maintaining physiological homeostasis, as well as damage caused by aggressive behavior (reviewed in Taranger et al. 2010).

The objective of the two present experiments was to examine how smoltification (gill NKA activity, plasma Na, Cl, osmolarity) and maturation (plasma 11‐ketotestosterone (11‐KT), gonadosomatic index (GSI), testicular histology, plasma Ca) codevelop in farmed Atlantic salmon, to explore potential developmental conflicts.

## Materials and Methods

### Experiment 1

#### Fish stock

Atlantic salmon fry of the Aquagen strain were first‐fed under continuous light and elevated temperature (12–13°C) in the spring of 2009. When the ambient natural water temperature had reached 12°C, around summer solstice, the temperature was changed to ambient, and the photoperiod switched from continuous light to simulated natural photoperiod (60° N, 5° E) on 01 October 2009.

#### Experimental conditions

**Figure 1:**
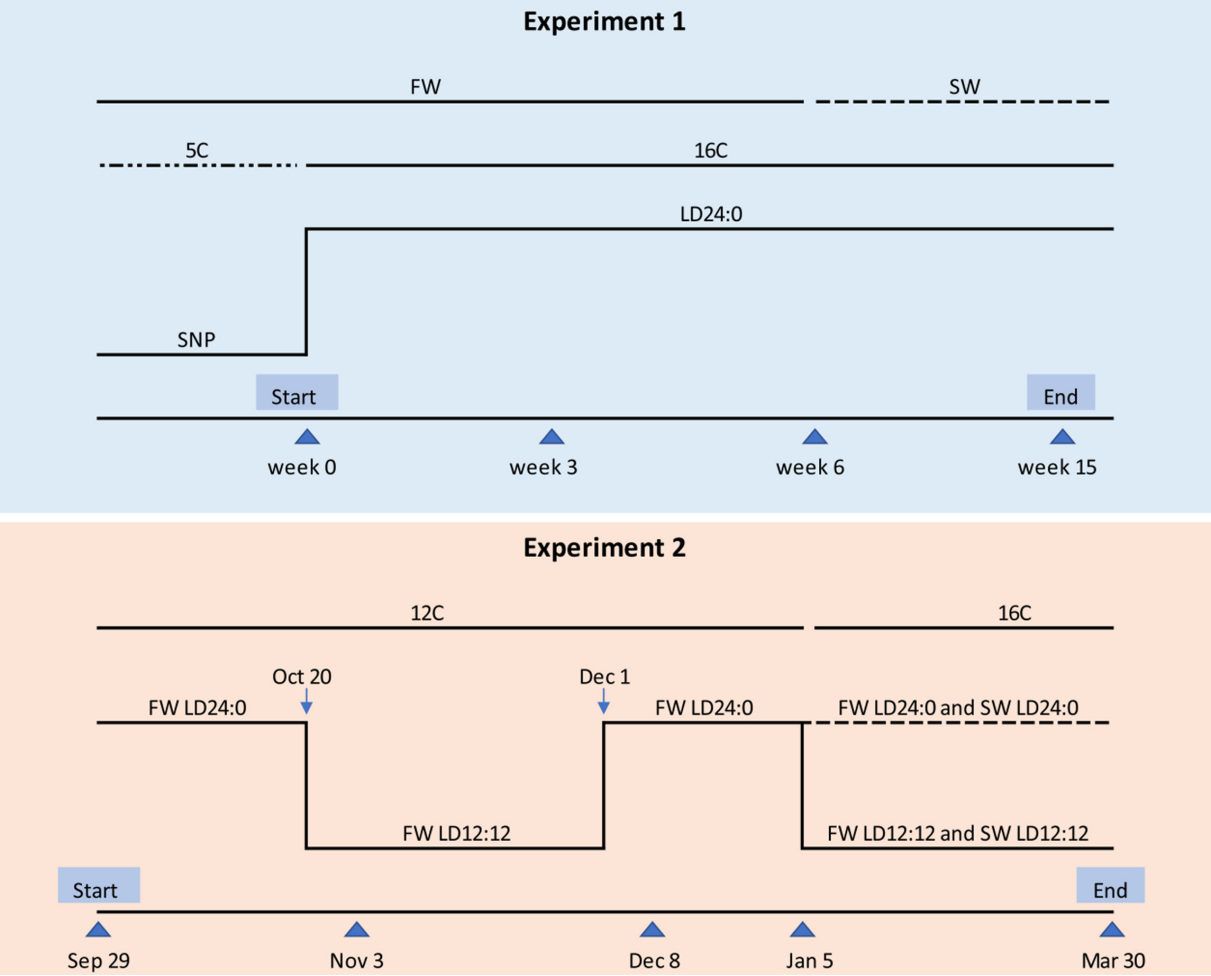
Experimental design. In experiment 1, the rearing environment was changed from ambient temperature and simulated natural photoperiod (SNP) to 16°C and LD24:0 on 12 February (week 0). Samples on week 6 were collected 24 h after change from freshwater (FW) to seawater (SW). In experiment 2, fish held at LD24:0 were changed to LD12:12 on 20 October followed by LD24:0 from 01 December to induce smoltification. On 05 January, the rearing water was changed from 12°C FW to either 16°C FW or SW, and four different treatment groups were created: LD24:0 FW, LD24:0 SW, LD12:12 FW, and LD12:12 SW. Samples were collected on the dates indicated by arrowheads.

On 09 February 2010, 105 Atlantic salmon presmolts (197 g) were randomly distributed between three 1 × 1 m tanks (35 fish per tank) under simulated natural photoperiod (60° N, 5° E) and natural temperature (5°C). On 12 February, the photoperiod was switched to LD24:0, and the temperature gradually increased from 5°C to 16°C over a 5‐day period. The salinity was changed from 1.7 ppt to 34.5 ppt on 24 March. Mean water temperature was 16°C (range 15.7–16.7°C) in freshwater, and 16.8°C (range 16.0–17.2°C) in seawater. The experimental design is shown in Figure 1.

#### Tissue sampling

Fish were sampled on 12 February (week (w) 0), 04 March (w 3), 25 March (w 6, 24 h after change from fresh‐ to seawater), and 25 May (w 15). On the first sampling (w 0), five fish were sampled per tank. On the remaining sampling points (w 3–15), 10 fish were sampled per tank. The fish were euthanized (Finquel vet., Scanvacc AS, 0.5 g L^−1^), sexed and measured for fork length (cm), body and gonad weight (g), and sampled for blood. Testis tissue was fixed in 4% buffered formalin. Blood was centrifuged and plasma stored at −80°C until further analysis.

#### Testis histology

**Figure 2:**
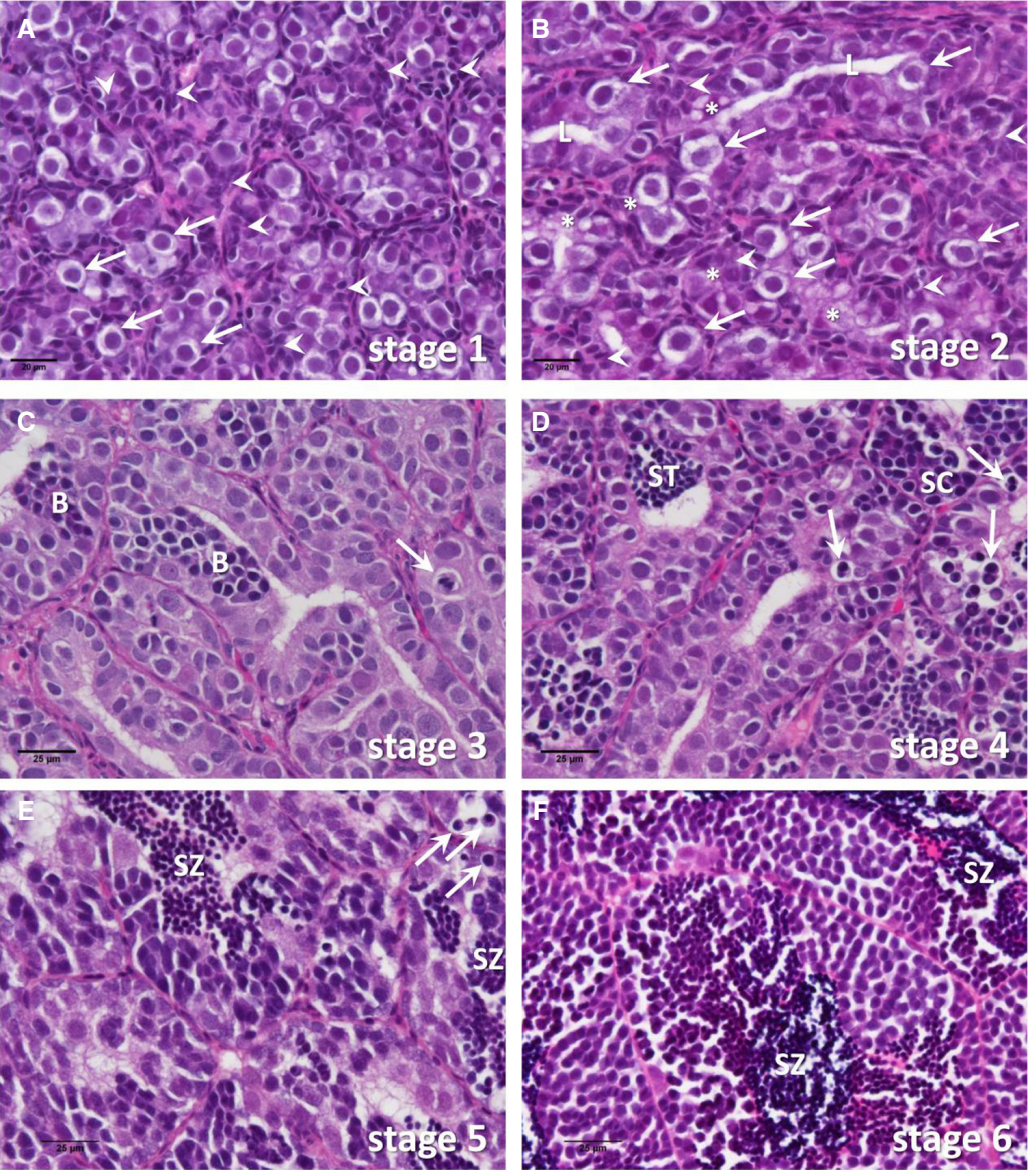
Representative examples of the testicular histology of Atlantic salmon in different stages of pubertal development. The bars in the lower left corners indicate 20 or 25 *μ*m. (A) Stage 1 testis containing type A spermatogonia (arrows) as furthest developed germ cell type. The spermatogenic tubules are compact and contain several groups of Sertoli cell nuclei (arrowheads), belonging to Sertoli cells apparently not contacting germ cells yet. (B) Stage 2 testis containing type A spermatogonia (arrows) as furthest developed germ cell type. Some spermatogenic tubules show lumina (L). Groups of Sertoli cell nuclei apparently not contacting germ cells are still frequent (arrowheads). In the cytoplasm of many Sertoli cells, vacuoles of varying size generate a “foamy” appearance (asterisk). (C) Stage 3 testis containing type A and type B spermatogonia (B). Sertoli cells not contacting germ cells are less frequent. Lumina are seen in most tubuli. Apoptotic germ cells (arrow) appear in some tubuli. (D) Stage 4 testis containing spermatocytes (SC) and/or spermatids (ST) as the furthest developed germ cell type. Apoptotic germ cells (arrow) are regularly seen. (E) Stage 5 testis containing spermatozoa (SZ) as the furthest developed germ cell type but earlier germ cell generations are still prominently present; apoptotic germ cells (arrow) become less frequent. (F) Stage 6 testis containing spermatozoa (SZ) as the furthest developed germ cell type that is present in large numbers in all tubuli; apoptotic germ cells are much less frequent

The following germ cell types were differentiated, according to criteria described previously (Melo et al. 2014): type A spermatogonia, type B spermatogonia, spermatocytes, spermatids, and spermatozoa. Moreover, testis tissue starting to mature can contain many Sertoli cells that are not (yet) in contact with germ cells, so‐called “free” Sertoli cells (Franca et al. 2016). Also, Sertoli cells can contain several small vacuoles, leading to the impression of a foamy cytoplasm. The presence of free Sertoli cells and of Sertoli cells with a foamy cytoplasm was noted. We also recorded the presence of a lumen in the spermatogenic tubules, which coincides with the presence of foamy Sertoli cells. Finally, in particular during the rapid growth phase of the testis, apoptotic germ cells were observed frequently and their presence was noted. These observations were used to define the following six stages of testis development (Fig. 2):

1. Type A spermatogonia as the furthest developed germ cell type; many free Sertoli cells; lumen not visible in spermatogenic tubules.
2. Type A spermatogonia as the furthest developed germ cell type; many free Sertoli cells; foamy Sertoli cell cytoplasm; lumen formation starts.
3. Type B spermatogonia as the furthest developed germ cell type; low number of free Sertoli cells.
4. Spermatocytes and/or spermatids as the furthest developed germ cell type; apoptotic germ cells regularly present; no free Sertoli cells.
5. Spermatozoa as the furthest developed germ cell type but earlier germ cell generations were still prominently present; apoptotic germ cells regularly present.
6. Spermatozoa as the furthest developed germ cell type present in large numbers in all tubuli; apoptotic germ cells much less frequent.

#### Plasma analysis

Steroids were extracted from blood plasma by a method modified from Pankhurst and Carragher (1992). Briefly, plasma samples (100 *μ*L) were mixed with 1 mL ethyl acetate, vortexed for 20 sec and centrifuged for 3 min at 417 rcf and 4°C. The organic phase was collected with a Pasteur pipette and the aqueous phase was extracted once more with 1 mL of ethyl acetate. The extracts were evaporated in a Speed Vac centrifuge (Savant 1000, USA), and dissolved in 1 mL buffer (phosphate 0.1 mol L^−1^ pH 7.4, 0.4 mol L^−1^ NaCl, 1 mmol L^−1^ EDTA) by heating (60°C for 10 min). The extracted and dissolved steroids were stored at −20°C until analysis by enzyme‐linked immunosorbent assay (ELISA) (Cuisset et al. 1994). ED80 and ED20 were 0.04 ng mL^−1^ and 1.00 ng mL^−1^ and detection limit of the assay was 0.005 ng mL^−1^. Samples with a binding >ED80 were outside of the linear portion of the standard curve, and were given a value of 0.05 ng mL^−1^, corresponding to the detection limit corrected for dilution after plasma extraction. Samples with a binding <ED20 were diluted further and re‐run until they fitted in the linear part of the standard curve.

Internal standards for 11‐KT were prepared from mature male Atlantic salmon plasma extracted as described above. The accepted interassay coefficient of variation was 10%; assays with higher deviation of the internal standard were re‐run. The intraassay coefficient of variation was 6.2% for 11‐KT (*n* = 10). Acetylcholine esterase‐labeled tracers and microplates precoated with monoclonal mouse antirabbit IgG were supplied by Cayman Chemicals (USA). Anti‐11‐KT was a kind gift from D. E. Kime, Sheffield University, UK, with details on cross‐reactivity given by Cuisset et al. (1994). Standard steroids were purchased from Sigma Aldrich (Sigma reference standards).

Plasma ion levels (Na, Cl) were detected by ion selective electrodes using a 9180 Electrolyte Analyser (Roche Diagnostics, Minnesota, USA). Plasma osmolarity was determined by freeze point determination (Fiske micro‐osmometer Model 210, Norwood, Mass, USA).

#### Gill NKA activity

Gill Na^+^, K^+^‐ATPase enzyme (NKA) activity was determined by the method of McCormick (1993). Briefly, this kinetic assay utilizes the hydrolysis of ATP, which is enzymatically coupled to the conversion of NADH to NAD^+^ by pyruvate kinase and lactic dehydrogenase with or without the addition of ouabain, a specific inhibitor of NKA. Readings were done at 340 nm for 10 min at 25°C and enzyme activity is expressed as *μ*mol ADP*mg protein^−1^*h^−1^.

### Experiment 2

The materials and methods and results on sexual maturation (GSI, testis histology, pituitary mRNA expression of *fshb*,* lhb*, and *gnrh4*) for this experiment have been published earlier (Melo et al. 2014). Here, we show yet unpublished data on smoltification physiology (gill NKA activity, *nka α*1a and *α*1b subunit mRNA expression), in order to examine the interaction of maturation and smoltification in male salmon.

In brief, the fish were reared at 12°C under LD24:0 for 28 weeks from first feeding, followed by LD12:12 for 6 weeks and LD24:0 for 4 weeks to complete smoltification. Finally, the water temperature was raised to 16°C, and the fish were divided into four subgroups with fresh‐ or seawater, and LD12:12 or LD24:0. The experimental design is shown in Figure 1.

For measurement of gill *nka α*1a and *α*1b subunit mRNA expression, total RNA was isolated from 30 mg gill tissue by phenol‐chloroform extraction using TRI Reagent^®^ (Sigma, St. Louis, MO, USA) according to the manufacturer's guideline. Total RNA quantity was determined using a ND‐1000 spectrophotometer (NanoDrop Technologies, NC, USA). RNA integrity was analyzed on the Agilent 2100 expert bioanalyzer (Agilent technologies, Santa Clara, CA, USA). cDNA was generated from 2 *μ*g total RNA in conjunction with Oligo d (T15–18) using the SuperScript III kit (Invitrogen, Carlsbad, CA, USA) as described by the manufactures.

Gill mRNA expression was analyzed on an SDS 7900HT Real‐Time PCR 384 well plate system (Applied Biosystems, Foster city, CA, USA). All q‐PCR assays were performed using 3 *μ*L diluted cDNA, 200 nmol L^−1^ of each primer and IQ SYBR Green Supermix (Bio‐Rad Laboratories, Hercules, CA) in a total reaction volume of 10 *μ*L. The thermal cycling protocol consisted of 2 min at 50°C, 10 min at 95°C followed by 40 cycles at 95°C for 15 sec and 60°C for 1 min. Melt curve analysis verified that the primer sets for each q‐PCR assay generated one single product and no primer‐dimer artifacts. cDNAs was diluted 1:60 and all samples were run in duplicate using primers previously published (Nilsen et al. 2007, 2010). For each assay, triplicate fivefold cDNA dilution series were used to determine amplification efficiencies (*E*) calculated as the slope from the plot of log RNA concentration versus threshold cycle (Ct) values using the following formula: E = 10^(−1/slope)^. This efficiency was used to correct for difference in amplification efficiency when calculating gene expression according to Pfaffl (2004). Elongation factor 1 alpha (*ef1a*) was used as an endogenous reference gene (Olsvik et al. 2005) and did not change over time or differ between treatments in the present study.

### Statistics and calculations

The gonadosomatic index (GSI) was calculated as GSI = (gonad weight*100) body weight^−1^.

The condition factor (CF) was calculated as CF = (body weight*100) (fork length^3^)^−1^.

The data were analyzed using Statistica version 12 (StatSoft, Inc., 2300 East 14th Street, Tulsa, Oklahoma, USA). Results are shown as means with their standard errors. Sampling point differences within different parameters within sexes were tested by one‐way ANOVAs. Sampling point and sex differences within different parameters were tested by two‐way ANOVAs. Significant ANOVAs were followed by Newman–Keuls post hoc tests to detect possible differences between sampling points. Possible significant correlations between measured parameters within sampling points were tested by Product‐Moment and Partial Correlations. *P* < 0.05 was classified as statistically different.

## Results

### Experiment 1

There was no mortality during the study period.

#### Male maturation

**Figure 3:**
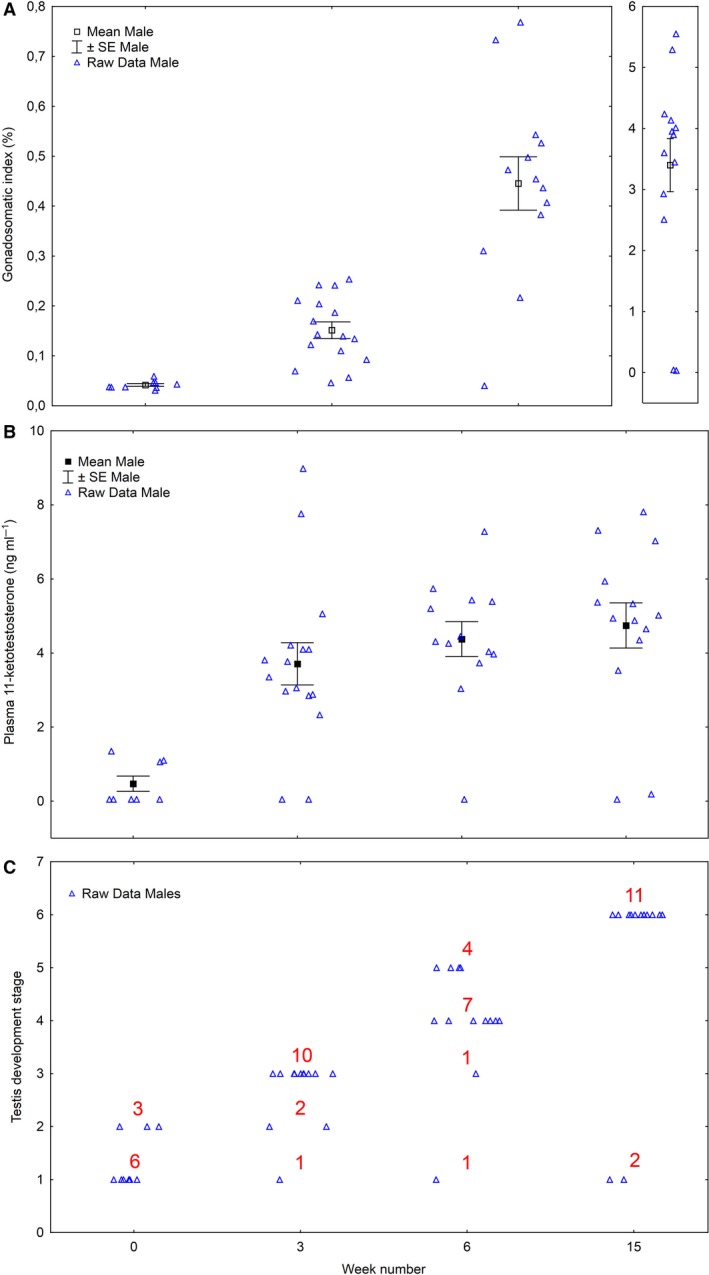
Development in maturation parameters (raw data blue triangles, and mean ± SE) over time among salmon males subjected to 16°C and continuous light for 15 weeks, Experiment 1. Water was changed from fresh‐ to seawater on week 6. Values on weeks 6 is 24 h after change in water salinity. Gonadosomatic index (A), plasma 11‐ketotestosterone (B), testis developmental stage – histology (C). Numbers in red (C) describe number of individuals per testis developmental stage.

At the initial sampling (*n* = 9), before the change to 16°C and LD24:0, all males had low gonadosomatic indexes (GSI) (min 0.031, max 0.059, mean 0.041, SE ± 0.002) and plasma 11‐KT levels (min ≤ 0.05, max 1.35, mean 0.47, SE ± 0.18). Histologically, six males were in stage 1, 3 in stage 2 (Fig. 3A–C).

After 3 weeks in the stimulating environment (16°C and LD24:0), only one of 13 males analyzed had a GSI value (0.045) in the range found in week 0, accompanied by low plasma 11‐KT levels and a stage 1 testis (Fig. 3A–C). The 12 other males all had GSI values higher than the highest value found at week 0 (GSI > 0.059; min 0.070, max 0.24, mean 0.16, SE ± 0.02). These maturing males also had increased plasma 11‐KT levels (min 2.33, max 7.76, mean 3.85, SE ± 0.43), and histologically, two showed stage 2 testis, and ten showed stage 3 testis. Notably, males with stage 2 testis at week 3 had higher plasma 11‐KT levels (week 0; 0.05 and 0.05 ng L^−1^ vs. week 3; 2.85 and 3.77 ng L^−1^) and GSI values (week 0; 0.04 and 0.06 vs. week 3; 0.07 and 0.14) than those with type 2 testis at week 0 (in one fish with GSI 0.046 and classified as stage 2 in week 0, the plasma sample was lost and 11‐KT levels not analyzed). In week 3, 13 males were sampled from the same population as the fish in the present experiment were collected from. These were still on natural temperature and photoperiod. Their GSI values (min 0.030, max 0.050, mean 0.038, SE ± 0.002) and 11‐KT levels (min 0.05, max 1.30, mean 0.28, SE ± 0.12) were within the range found for the experimental fish sampled on week 0; however, testis tissue was not analyzed histologically.

After 6 weeks under stimulating conditions, only one of the 13 males analyzed had a GSI value and 11‐KT level as low as found in week 0 and stage 1 testis (Fig. 3A–C). One male had a GSI value (0.22) similar to those found in week 3, with an 11‐KT level of 5.39 ng L^−1^ and a testis in stage 3. The remaining 11 males showed GSI values of 0.31–0.77 and testis tissue in stage 4 (*n* = 7) or 5 (*n* = 4) and 11‐KT plasma levels ranging between 3.04 and 7.28 ng L^−1^.

In the final sampling (after 15 weeks on the stimulating regime), two of the 13 males analyzed had low GSI values, low 11‐KT plasma levels and stage 1 testis, similar to those found at week 0 (Fig. 3A–C). The 11 other males had testes in stage 6, GSI values between 2.51 and 5.55 (mean 4.01, SE ± 0.27), and 11‐KT levels ranging between 3.53 and 7.81 ng L^−1^ (mean 5.48, SE ± 0.40). There was a positive linear regression (*R* ^2^ = 0.65, *P* < 0.0001) between plasma 11‐KT level and the testicular stages of development (1–6) when including all fish and sampling points.

**Figure 4:**
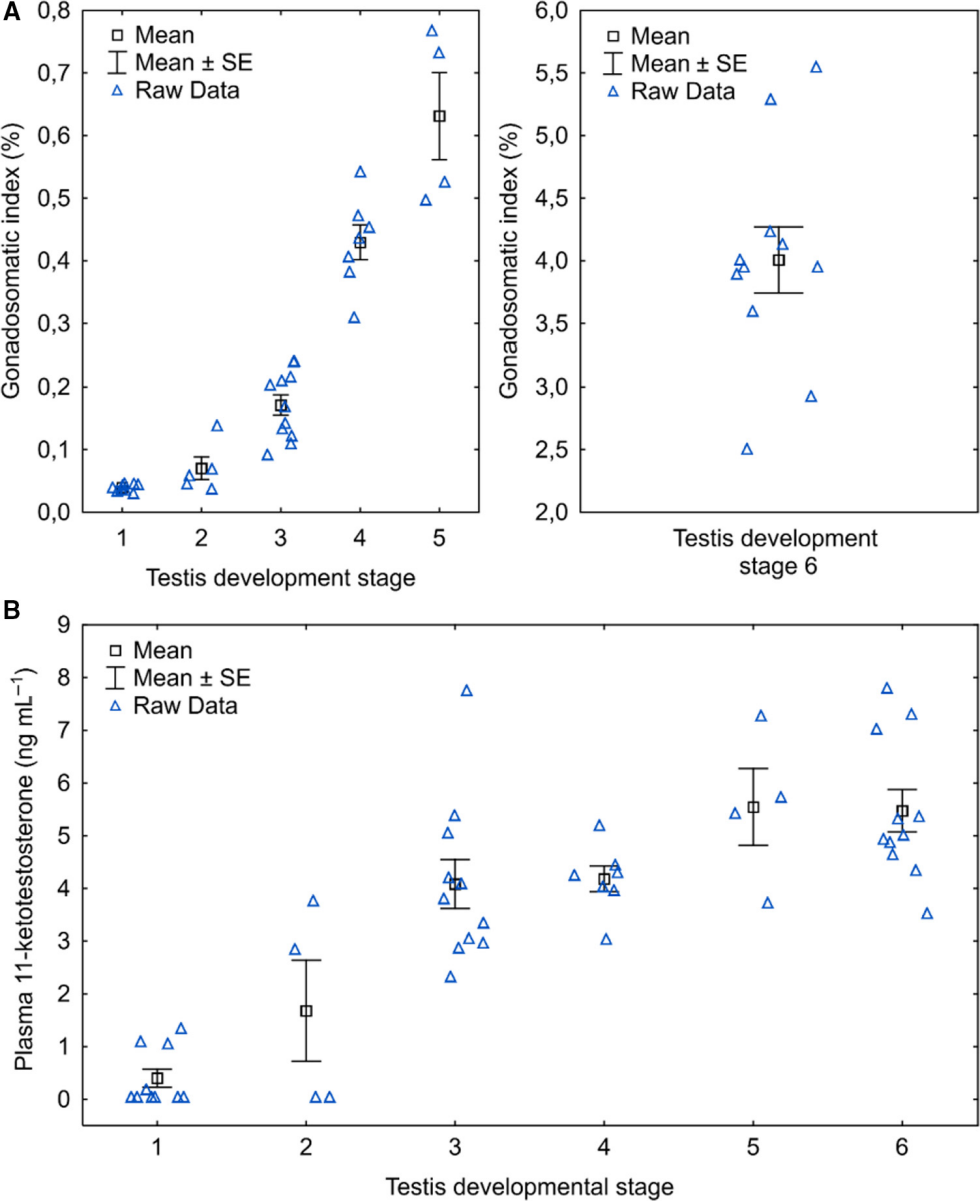
Males in experiment 1. Raw data for gonadosomatic index (A) and plasma 11‐ketotestosterone (B) for each testis development stage.

Raw data for GSI and 11‐KT for each histological stage are shown in Figure 4.

#### Female maturation

**Figure 5:**
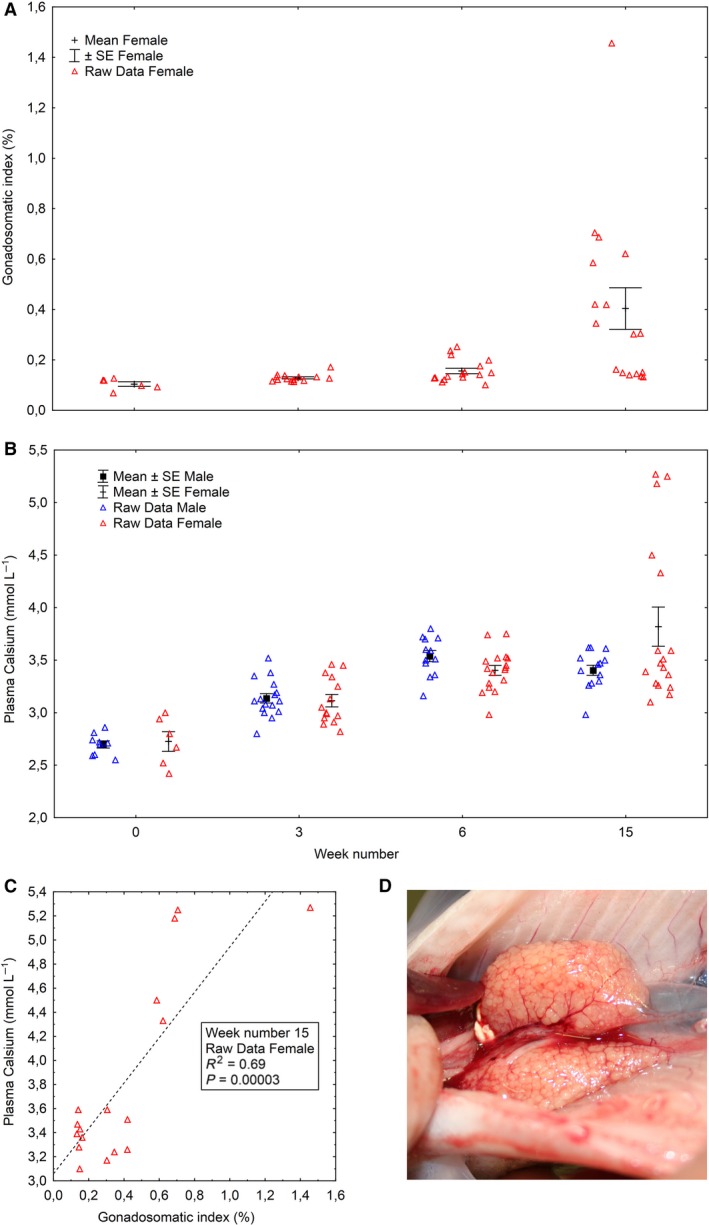
Development in maturation parameters (raw data red triangles, and mean ± SE) over time among salmon females subjected to 16°C and continuous light for 15 weeks, Experiment 1. Water was changed from fresh‐ to seawater on week 6. Values on weeks 6 is 24 h after change in water salinity. Gonadosomatic index (A), plasma calcium (B), correlation between plasma calcium and gonadosomatic index in week 15 (C), photo of ovary with highest gonadosomatic index in week 15 (1.45%) (D).

In females, the GSI was low and stable in week 0 and 3 (Fig. 5A). In week 6, 13 of the 17 analyzed females had a GSI that was within the range recorded in week 0 and 3. At the final sample (after 15 weeks on the stimulating regime), seven of the 17 analyzed females had GSI values that were within the rage recorded in weeks 0, 3, and 6 (Fig. 5A). Plasma total Ca was similar among females and males in weeks 0, 3, and 6, but significantly higher (two‐way ANOVA, *P* < 0.05) in females than males in week 15 (Fig. 5B). There was also a significant positive correlation between plasma total Ca and GSI in females at this sampling (Fig. 5C). This was not observed at any other time point (data not shown).

#### Secondary sexual characteristics

**Figure 6:**
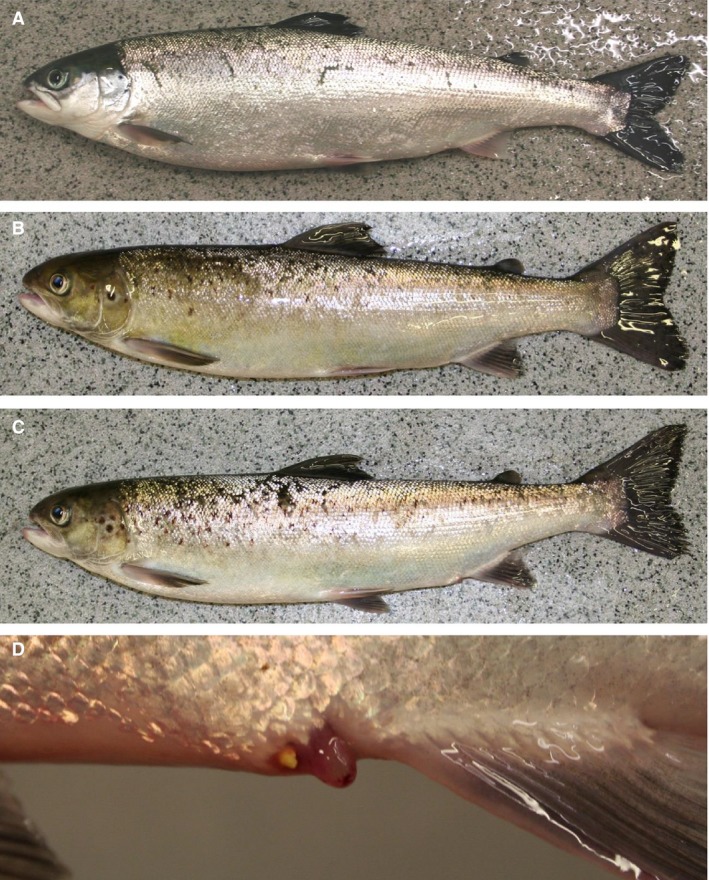
Secondary sexual characteristics, Experiment 1. Lateral photographs taken after 15 weeks with 16 °C and continuous light. Water was changed from fresh‐ to seawater on week 6. Immature female (A), mature male (B), maturing female (C), the enlarged gonopore of a maturing female (D).

Males and females had similar secondary sexual characteristics; the males developed a “female mimicry” with a head with no kype (Fig. 6B). The most obvious sexual dimorphisms were more elongated pectoral fins in maturing males (Fig. 6B), and enlarged gonopores in maturing females (Fig. 6C and D). An immature fish is shown in Figure 6A for comparison.

#### Growth

**Figure 7:**
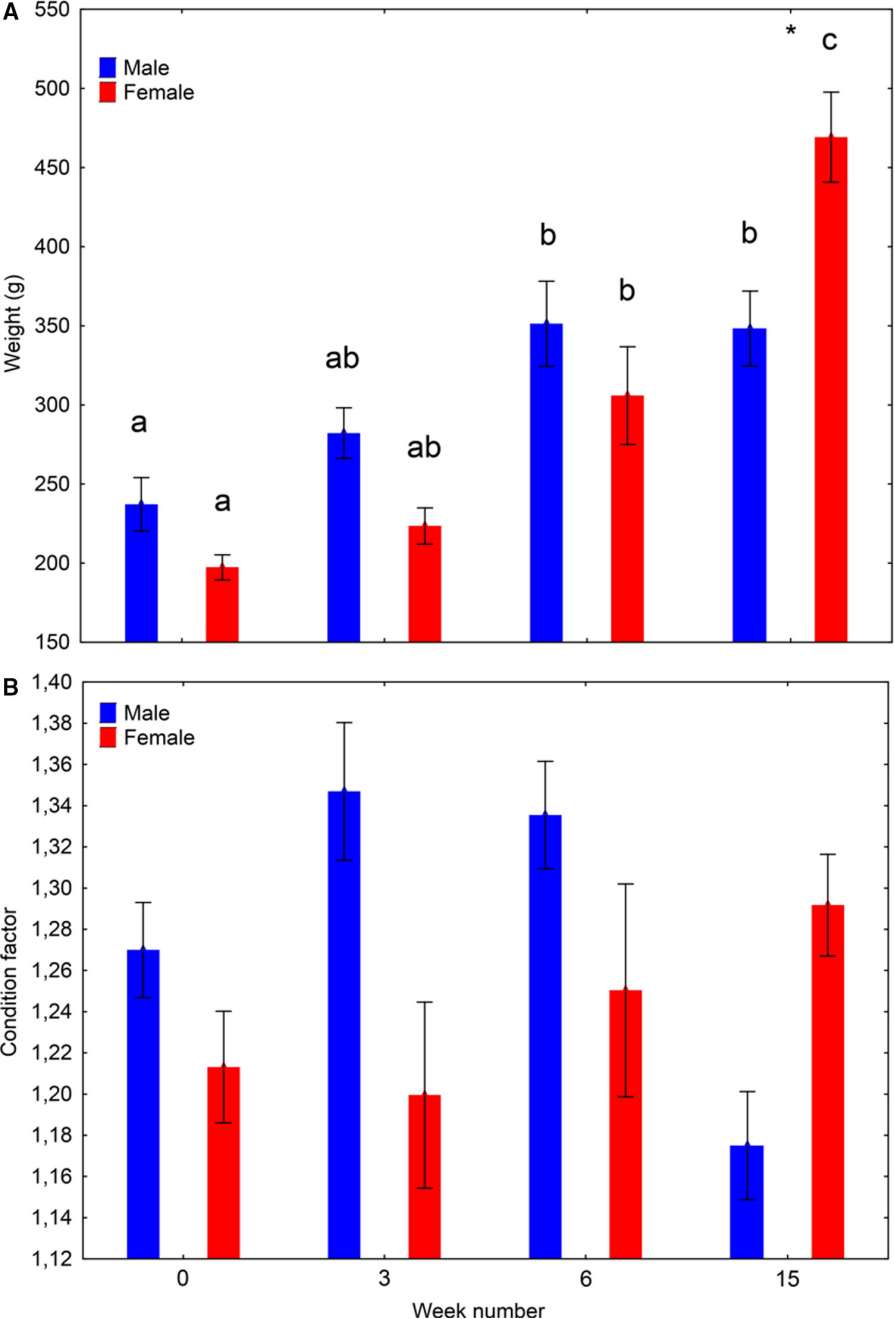
Somatic growth (mean ± SE), Experiment 1. Development in body weight (A) and condition factor (B) over time among salmon males and females subjected to 16°C and continuous light for 15 weeks. Water was changed from fresh‐ to seawater on week 6. Different lowercase letters indicates significant differences (*P* < 0.05) within sexes between sampling points. “*” indicates significant differences between sexes within sampling point.

There was a significant effect (two‐way ANOVA) of time (week number) (*P* < 0.00001) and a time*sex interaction (*P* = 0.0004) on body weight. In males, the weight increased until week 6, and then leveled out, whereas in females, it increased further until the terminal sampling in week 15 (Fig. 7A). Females were significantly heavier than males in week 15.

There was a significant interaction (two‐way ANOVA, *P* < 0.05) between time and sex on condition factor, but there were no significant differences over time within sexes, or between sexes at each time point (Fig. 7B).

#### Osmoregulation

**Figure 8:**
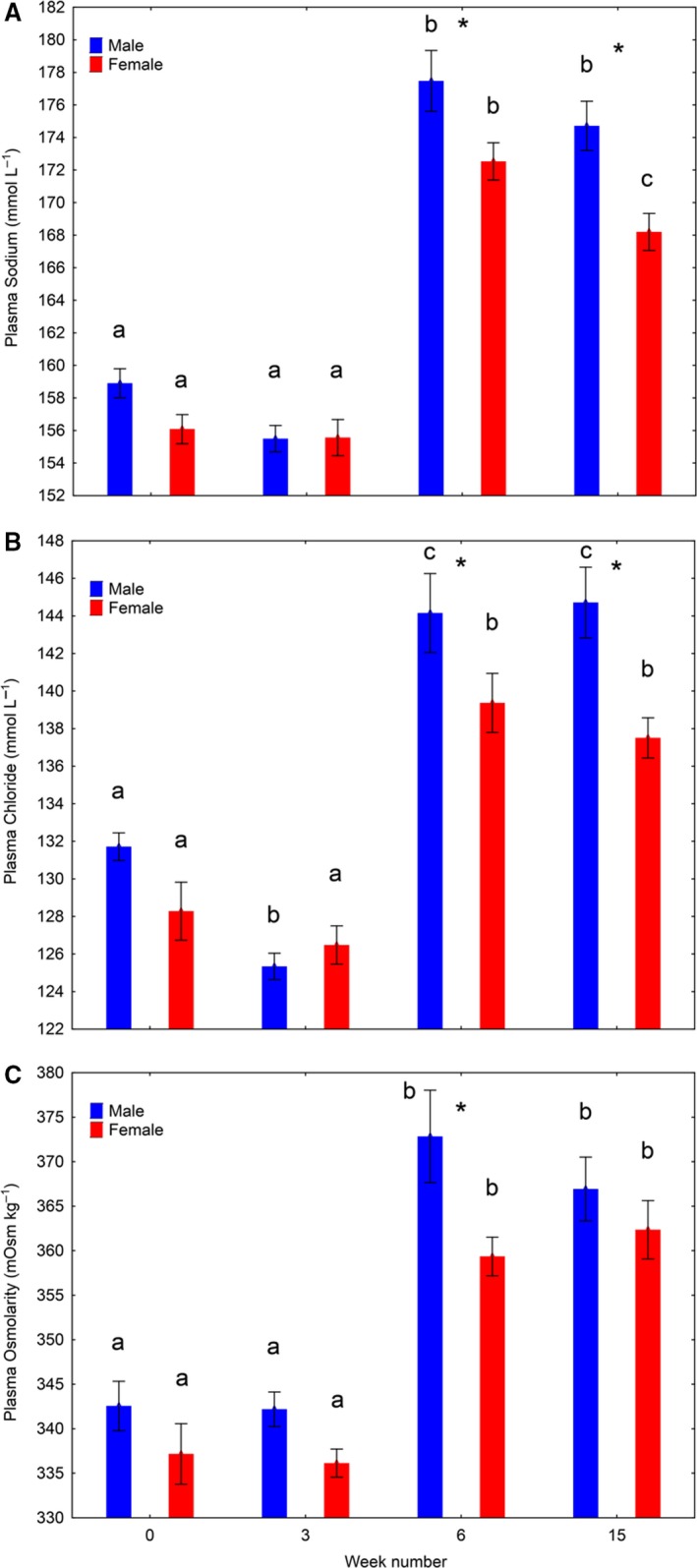
Plasma ion balance (mean ± SE), Experiment 1. Development in plasma sodium (A), chloride (B), and osmolarity (C) over time among salmon males and females subjected to 16°C and continuous light for 15 weeks. Water was changed from fresh‐ to seawater on week 6. Values on weeks 6 is 24 h after change in water salinity. Different lowercase letters indicates significant differences (*P* < 0.05) within sexes between sampling points. “*” indicates significant differences between sexes within sampling point.

There was a significant effect (two‐way ANOVA, *P* < 0.01) of time (week number) and sex on plasma Na, Cl, and osmolarity, and also a significant interaction (*P* < 0.05) between time and sex on plasma Na and Cl. In both sexes, plasma Na, Cl, and osmolarity was significantly higher in week 6 and 15 compared to week 0 and 3 (Fig. 8A–C). In females, we also found a significant reduction in plasma Na between weeks 6 and 15 (Fig. 8A). Males had significantly higher plasma Na, Cl, and osmolarity than females in week 6, and significantly higher plasma Na and Cl in week 15 (Fig. 8A–C).

**Figure 9:**
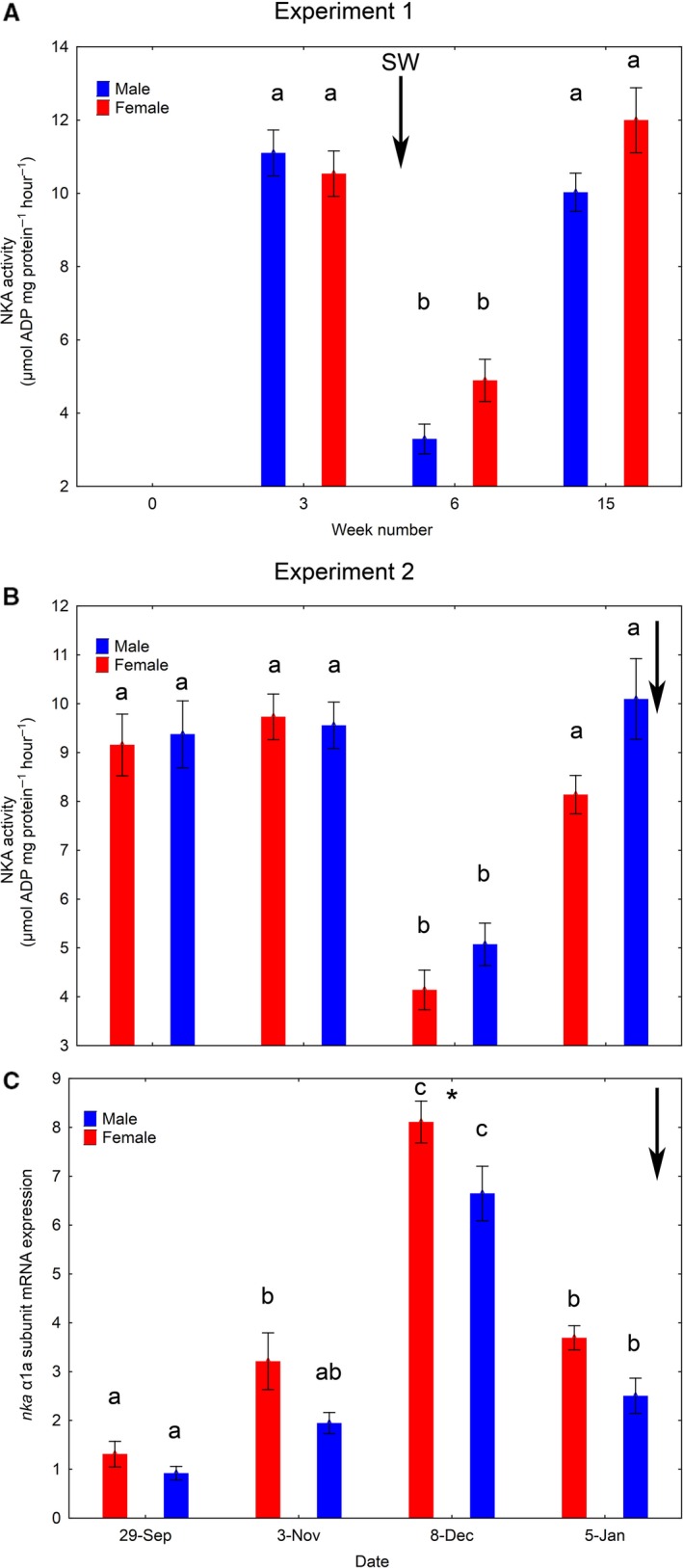
Osmoregulation (mean ± SE). Experiment 1 (A): Development in gill Na^+^, K^+^‐ATPase enzyme (NKA) activity over time among salmon males and females subjected to 16°C and continuous light for 15 weeks. Water was changed from fresh‐ to seawater on week 6. Values on weeks 6 is 24 h after change in water salinity. Experiment 2 (B and C): Development in gill NKA activity (B) and gill *nka α*1a subunit mRNA expression (C) over time among salmon males and females reared under LD12:12 for 6 weeks followed by continuous light for 4 weeks in 12°C freshwater. Black errors indicate when water salinity was changed from fresh to salt. Different lowercase letters indicates significant differences (*P* < 0.05) within sexes between sampling points. “*” indicates significant differences between sexes within sampling point.

There was a significant effect (two‐way ANOVA, *P* < 0.00001) of time on gill NKA activity (Fig. 9A). The enzyme activity was significantly lower in week 6 compared to weeks 3 and 15 in both sexes (Fig. 9A).

### Experiment 2

#### Sexual maturation and gill physiology

There was a significant effect (two‐way ANOVA, *P* < 0.00001) of time point on gill NKA activity. Gill NKA activity was already high in both males and females on 29 September, when the fish had been on continuous light since first feeding (Fig. 9B). The photoperiod was changed to LD12:12 on 20 October, and back to LD24:0 on 01 December. The gill NKA activity was as follows: (1) still high on 03 November, 14 days after change to LD12:12; (2) significantly reduced on 08 December, 7 days after shift back to LD24:0; (3) high again on 05 January, 35 days after shift back to LD24:0 (Fig. 9B). There was a significant effect (two‐way ANOVA) of both sex (*P* = 0.0002) and time point (*P* < 0.00001) on gill *nka α*1a subunit mRNA expression. Females generally had higher values than males, with a significant difference on 08 December (Fig. 9C). With time, in both sexes, there was a significant increase in the gill *nka α1a* transcript levels between 29 September and 08 December, and a significant decrease between 08 December and 05 January (Fig. 9C). There were no significant effects of sex or time point on gill NKA *α*1b subunit mRNA expression (sexes pooled mean values per sampling point: 0.86, 0.80, 0.78, and 1.04).

On 05 January, when the fish were ready for seawater transfer, four of the analyzed males were classified as immature, and four as maturing based on GSI and a number of other parameters (see Melo et al. 2014 for details). Regarding the gill NKA activity (immature 11.1 ± 0.3, maturing 9.1 ± 1.5 *μ*mol ADP mg protein^−1^ h^−1^) of these eight males, there was no significant difference (one‐way ANOVA, *P* = 0.238) between immature and maturing individuals. Gill mRNA expression was not analyzed in two of the four immature males; a statistical testing for differences in gill mRNA expression between immature and maturing males was not possible.

#### Secondary sexual characteristics

**Figure 10:**
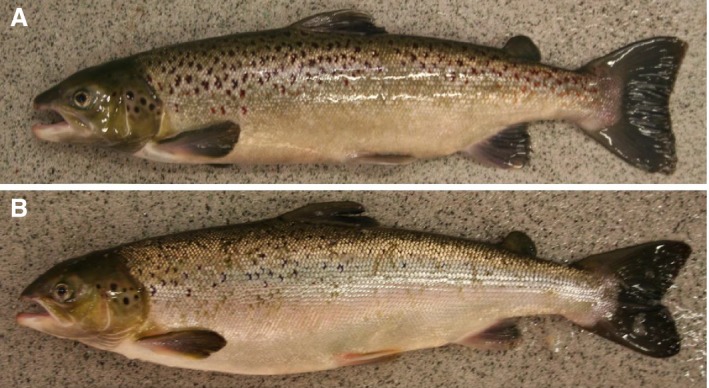
Secondary sexual characteristics, Experiment 2. Lateral photographs taken at termination of the experiment, after the fish had been under either LD12:12 (A) or LD24:0 (B) for 12 weeks in 16°C fresh‐ or seawater. There was no observed salinity effect.

At termination of the experiment, on 30 March, after the fish had been under either LD12:12 or LD24:0 for 12 weeks in 16°C fresh‐ or seawater, the maturing males showed some degree of kype formation (Fig. 10A and B). Also, at the same time point, maturing males that had been under LD12:12 had a stronger skin pigmentation than maturing males at LD24:0, regardless of water salinity (Fig. 10A and B).

## Discussion

Several studies on Atlantic salmon investigated physiological changes during smoltification and maturation, how environmental conditions affect these changes and how that may serve different maturational strategies, but this is the first study to show the physiological and phenotypical changes that accompany maturation and smoltification occur in parallel.

We found that the maturational response to a stimulatory environment in salmon males is very rapid. This response probably involved activation of the brain–pituitary system and elevated release of pituitary gonadotropin, which in turn initiated spermatogenesis by modulating the production of hormones/growth factors to promote germ cell development (reviewed by Zohar et al. (2010), Levavi‐Sivan et al. (2010), Schulz et al. (2010)). Although we did not directly study brain or pituitary factors in experiment 1, the four to eightfold increases in GSI, gonad weight and plasma 11‐KT levels in just 3 weeks are clearly indicative of strong signals promoting male puberty. In experiment 2, maturing males had higher plasma 11‐KT concentrations and pituitary mRNA transcript levels of *fshb*,* lhb*, and *gnrh4* than immature males on 05 January (Melo et al. 2014). The currently used high water temperatures may have increased the developmental speed of the maturation process, whereas similar changes would require longer time to occur in nature with lower water temperatures.

The present study is the first to report the early changes during induced maturation in Atlantic salmon males. In a previous study (Melo et al. 2014) that provided the samples analyzed for the osmoregulatory parameters in experiment 2, initiation of the early changes during induced maturation in Atlantic salmon males was missed since maturation had commenced in half of the males before the water temperature was raised to 16°C. Three males in the current experiment 1 showed stage 2 testis at the first sampling, that is, under natural photoperiod and temperature in February. Whether these males indeed already had started pubertal development spontaneously is not clear. In any case, these three males had lower plasma 11‐KT concentrations and GSI than fish with stage 2 testis at week 3, and the males that continued to be under nonstimulatory conditions (natural light and temperature) in week 3, showed plasma 11‐KT and GSI values as low as in the three stage 2 fish in week 0. This could indicate that the stage 2 testes in week 0 also represents an immature status. Future studies should investigate the early testicular changes in more detail, but also how the brain–pituitary system becomes activated. Regarding the pituitary gonadotropins, it is important to note that in salmonids, measurable plasma levels of luteinizing hormone (Lh) are restricted to the final stages of germ cell maturation when testicular growth has been completed (Gomez et al. 1999; Campbell et al. 2003). Accordingly, entry into puberty and progress through much of the rapid testicular growth phase is independent of Lh and instead regulated by Fsh. This renders studies into the regulation of Fsh production and release highly relevant for understanding how male pubertal development is triggered in salmonids. Such basic knowledge on the endocrine and molecular mechanisms triggering sexual maturation is a prerequisite for developing approaches to prevent precocious, unwanted puberty in fish.

**Figure 11:**
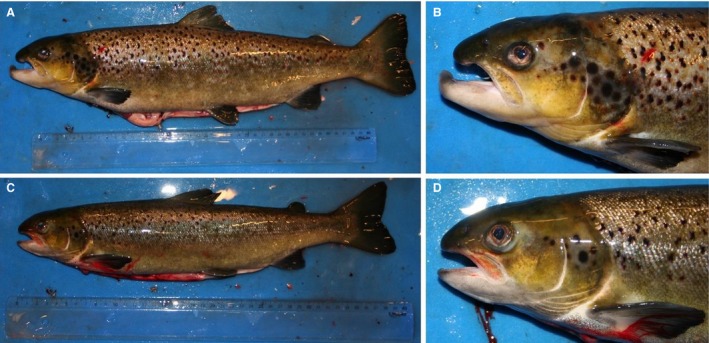
Lateral photographs of mature male Atlantic salmon from a separate study. Included to show the size phenotype relationship for male secondary sexual characteristics. Large animal (48.4 cm fork length) (A and B), small aninal (38.1 cm fork length) (C and D). The photographs were taken on 21 December 2010, and both animals were at the same stage of development (running milt), were from the same population, and had been reared in the same tank under natural light and temperature. Note the male (A and B) and female (C, D) phenotype.

Both experiments used relatively large fish and elevated temperature, and high levels of male maturation were observed. Indeed, both fish size and temperature are important risk factors for early maturation in Atlantic salmon males. Comparing different studies, there is an increasing level of male postsmolt maturity with: (1) increasing fish size, both when applying maturation stimulating environments (present study; Fjelldal et al. 2011; Melo et al. 2014), and under natural environmental conditions (Fjelldal et al. 2007); (2) increasing water temperature in the range 8.3–16°C (Fjelldal et al. 2011; Imsland et al. 2014; Sambraus 2016). Size is also important for Atlantic salmon males that matured as parr to either re‐enter maturation or to smoltify the following spring (Berglund et al. 1992). Regarding male secondary sexual characteristics, the maturing males in experiment 1 were smaller and did not develop a kype on the lower jaw, whereas in experiment 2, where the maturing males were larger, they did develop a kype. Males in experiment 2 had progressed further into maturation and many males had reached spawning condition and spermatogenesis was completed (see Melo et al. 2014), whereas the males in experiment 1 were still in the testicular growth phase, and showed lower plasma androgen levels and many germ cell cysts that still had to complete spermatogenesis. Hence, the morphological differences may be linked to maturational stage and plasma androgen levels. However, maturing male postsmolts of around 250 g body weight may develop a “female mimicry” with a head with no kype, similar to precocious male parr (Fjelldal et al. 2011). Photographs of mature male Atlantic salmon from a separate study are included as supplementary material (Fig. 11). The photographs show a small mature male (38 cm fork length) with a female phenotype and a larger male (48 cm fork length) with a male phenotype, both from the same population and sampled at the same time point and stage of development (full maturity, running milt). Further studies are needed to elucidate the impact of size, androgen levels and maturity state on development of secondary sexual characteristics in male salmon.

Normally, a period of 350 day degrees (dC) is needed from the onset of the smoltification‐related increase in gill NKA activity until peak activity is reached (Handeland et al. 2004). The duration of the smolt window can be in the range 300–400 dC (Sigholt et al. 1998; Stefansson et al. 1998; McCormick et al. 1999; Handeland et al. 2004), the smolt window closes at ~550 dC after smoltification has been initiated. The patterns of development in gill NKA activity were similar in both experiments, high–low–high over time, and the fish physiologically smoltified–desmoltified–resmoltified in both experiments. The reason for the initially high NKA activity differed between the two experiments. In experiment 1, it was related to a shift from natural light and temperature to continuous light and 16°C (week3: 320 dC) during the winter, whereas in experiment 2, the fish had smoltified under constant environmental conditions with continuous light from first feeding (29 September and 03 November). The ensuing desmoltification was caused by moving out of the smolt window under constant environmental conditions in experiment 1 (week 6: 660 dC), and by a change from LD24:0 to LD12:12 in experiment 2 (08 December). Finally, resmoltification was associated with the exposure to seawater under continuous light in experiment 1 (week 15: 1600 dC), and the change from LD12:12 to LD24:0 in freshwater in experiment 2 (05 January). Taken together the results show that large smolts have a highly dynamic osmoregulatory physiology, and can adapt quickly to changes in the environment. Indeed, the observation that all males in experiment 1 – despite being desmoltified and maturing – survived transfer to full strength seawater and had regained high gill NKA activity, while continuing sexual maturation after 9 weeks in seawater, is compelling evidence for the strength of the salmon's homeostatic physiology. The following observations suggest that certain traits associated with the processes of smoltification and the start of puberty can occur in parallel without apparent conflicts: (1) plasma androgen levels and testis weight increased in parallel with high gill NKA activity in male smolts (experiment 1); (2) gill NKA activity between immature and maturing male smolts were similar (05 January – experiment 2); (3) gill NKA activity of male and female smolts were similar (week 3 – experiment 1, 05 January – experiment 2). On the other hand, pointing toward a possible conflict are the female‐biased transcript levels of gill *nka α*1a subunit mRNA (experiment 2). Reciprocal expression of FW‐type (*α*1a) and SW‐type (*α*1b) catalytic *nka* subunit is often reflected in low and high NKA activity, respectively (Nilsen et al. 2007, 2010). We have observed increased gill *nka* 1a mRNA levels that correspond with lower enzyme activity in sexually maturing grilse without any effect on the *nka* 1b subunit isoform (Nilsen et al. unpubl.), suggesting that these two isoforms may be differentially regulated by sex steroids. The biological significance of this finding is, however, not clear at present. In nature, the gill NKA activity decreases in sexually mature and river running Atlantic salmon (Persson et al. 1998), and gonadal steroids negatively affected gill NKA activity (Madsen and Korsgaard 1989; McCormick 1995) and hypoosmoregulation (Lundqvist et al. 1989) in salmonids. Furthermore, higher gill NKA activity was recorded in sterile compared to mature brook trout females (*Salvelinus fontinalus*) (Le Francois and Blier 2000). The current observations of increased gill NKA activity and resmoltification in maturing males with already elevated plasma 11KT in experiment 1 deviate from this pattern, as does entering puberty alongside with resmoltification and increased gill NKA activity in females. The lack of difference in gill NKA, but different plasma Na, Cl, and osmolarity, between males and females in seawater in experiment 1 may indicate the importance of other organs in the regulation of seawater adaptability during sexual maturation. In teleosts, the gill, intestine, and kidney coordinate ion and water transport to ensure osmotic homeostasis. Interestingly, the aquaporin‐8b isoform has been suggested to be a key water channel responsible for water uptake in the intestinal tract of Atlantic salmon in seawater (Tipsmark et al. 2010). If intestinal aquaporins are involved in the mechanisms underlying the higher plasma ions and osmolarity in males compared to females in seawater in the present experiment 1 remains to be clarified. In contrast to the present observation, equal plasma osmolarity, Na, and Cl were recorded in immature males and females, and mature male chinook salmon (*Oncorhynchus tshawytscha*) males after 91 days in salt water (Bernier et al. 1993).This may indicate that there are differences in the osmoregulatory system of genus *Oncorhynchus* and genus *Salmo*.

It is not necessary for domesticated Atlantic salmon to experience a shift in photoperiod to smoltify or mature sexually. For instance, Imsland et al. (2014) reared Atlantic salmon at LD24:0 and 10–12°C from first feeding in March 2001 to 16 g (June 2001) and continued at 12.7°C to 586 g in July 2002. Peak gill NKA activity was recorded in November 2001 and postsmolt maturation in 82% of the males in July 2002. Still, applying a simulated natural photoperiod entrained smoltification compared to LD24:0, and Melo et al. (2014) showed an entraining effect of photoperiod on postsmolt maturation in males when comparing LD24:0 and LD12:12; the latter accelerating the completion of testis maturation. It appears that both developmental processes can take place without changes in the photoperiod but that in case such changes do occur, they entrain developmental changes. This is supported by the current observation of a further progressed spermatogenesis (Melo et al. 2014) and more developed secondary sexual characteristics – stronger skin pigmentation – under LD12:12 compared to LD24:0 in experiment 2. With the industry's present initiative to produce large smolts, often in RAS systems, sexual maturation in males, but also in females, should be monitored carefully. Male postsmolt maturation has been a significant problem in Tasmanian Atlantic salmon aquaculture, resulting in a switch toward all‐female production (Thorpe 2004). However, based on this study, maturation in females is a potential problem as well, in particular in large smolts. The present female maturation, shown by increased GSI and elevated plasma Ca, was associated with physiological resmoltification, and did not involve a change in photoperiod or temperature but in salinity from fresh‐ to seawater. Whether the currently observed female maturation was related to size, salinity change, triggered by the (salinity change‐induced) physiological resmoltification, or by a combination of these conditions, is not known at present. Our observation that a small fraction of males did not enter puberty, despite being reared in a stimulating environment for as long as 15 weeks, is highly relevant for the current trend to produce larger smolts. A focus on this resistance to postsmolt maturation, with an emphasis on genetic/epigenetic mechanisms and next generation effects, is knowledge‐building research for an intensive and sustainable farming of Atlantic salmon in the future. In this context, the discovery of a genetic locus in the salmon genome, encoding the *vgll3* gene, seems relevant since naturally occurring allelic variants in this gene can explain 33–36% of age at maturity in salmon males (Ayllon et al. 2015). This opens new possibilities for selective breeding and should be addressed in future studies.

The main finding of the current study is that domesticated Atlantic salmon: (1) males and females can physiologically smoltify–desmoltify–resmoltify within a short period of time; (2) males can start maturation and smoltification simultaneously, but also have the capacity to upregulate gill NKA activity and resmoltify while maturation is ongoing and plasma 11KT elevated; (3) females can enter puberty and resmoltify simultaneously. This is compelling evidence for the strength of the homeostatic physiology, and highly dynamic osmoregulatory physiology in domesticated salmon.

## Conflict of Interest

None declared.
